# Genomic insights into population structure and conservation of wild olive (*Olea europaea* subsp. *cuspidata*) in Oman’s Dhofar and Hajar Mountains

**DOI:** 10.3389/fpls.2026.1813482

**Published:** 2026-05-13

**Authors:** Thuraiya Al Jabri, Boshra Ahmed Halo, Alastair Culham, Richard Ellis

**Affiliations:** 1 Botany and Conservation Department, Oman Botanic Garden, Muscat, Oman; 2 School of Biological Sciences, University of Reading, Whiteknights, Reading, United Kingdom; 3 School of Agriculture, Policy and Development, University of Reading, Agriculture, Earley Gate, Reading, United Kingdom; 4 Department of Plant Sciences, College of Agricultural and Marine Sciences, Sultan Qaboos University, Al-Khoud, Muscat, Oman

**Keywords:** genetic diversity, *Olea europaea* subsp. *cuspidata*, Oman, population structure, SNP markers, wild olive

## Abstract

**Background:**

Wild olive (*Olea europaea* subsp. *cuspidata*), one of the six subspecies of the olive complex worldwide, is an ecologically and economically important tree in the mountains of Oman. It is vulnerable to various biotic and abiotic stresses. Information on its genetic diversity is needed to support the conservation of wild olive in Oman.

**Results:**

A dual-genomic approach was employed, utilizing nuclear SNPs derived from Angiosperms353 target enrichment and plastid SNPs (cpSNPs) recovered from off-target reads. These markers were used to assess the genetic diversity and population structure of 48 wild olive accessions from the Dhofar, Eastern Hajar, and Western Hajar mountains of Oman. STRUCTURE, PCA, and DAPC analyses consistently revealed a clear north–south genetic split, with Dhofar populations forming a distinct and genetically isolated lineage, while populations from the Eastern and Western Hajar Mountains showed evidence of admixture and genetic connectivity. Five plastid haplotypes were identified, further supporting this phylogeographic structure. Analysis of molecular variance (AMOVA) indicated that most genetic variation occurred within populations (71.86%) rather than among populations (28.14%; ΦST = 0.2814), while regional divergence between northern and southern populations was higher (ΦRT = 0.528). The overall fixation index (F_ST_ = 0.3093) indicated moderate to high genetic differentiation, with the strongest differentiation observed between northern and southern populations.

**Conclusions:**

These findings highlight the genetic uniqueness and isolation of Dhofar populations, which should be considered a conservation priority. The results provide essential guidance for *in situ* and *ex situ* conservation strategies, including germplasm preservation and habitat restoration.

## Introduction

*Olea europaea* subsp. *cuspidata* (Wall. & G.Don) Cif. (wild olive) is one of six recognized subspecies within the olive complex (Green et al., 1989). This subspecies is geographically and ecologically distinct from its Mediterranean relatives (Zohary, 1995), occurring across a broad range extending from southern and eastern Africa through the Arabian Peninsula to Iran, Pakistan, and India (Green, 2002). Its distribution may even reach the arid parts of Yunnan and Sichuan provinces in China (Green, 2002). Wild olive in Oman occurs at elevations between 900 and 2900 m (Ghazanfar, 2015) in the Western and Eastern Hajar Mountains in the north and the Dhofar Mountains in the south.

Wild olive is a monoecious, diploid species (2n = 46) primarily pollinated by wind (McGregor, 1976) and to a lesser extent by insects (Canale and Loni, 2010). Its fruits are consumed by several bird species and small mammals, which contribute to seed dispersal (Cuneo and Leishman, 2006; Abiyu et al., 2015). Although clonal reproduction has not been recorded in this subspecies, it has been observed in related taxa such as *O. europaea* subsp. *laperrinei* (Baali-Cherif and Besnard, 2005). Wild olive provides important ecological and socio-economic benefits, offering shade, forage, and edible fruits for local communities (Ghazanfar, 2018).

In Oman, wild olive trees are often in poor condition, showing little to no new growth, and the species was previously classified as of Least Concern (LC) but is now approaching Near Threatened (NT) status (Ghazanfar, 2015). Nevertheless, it was not listed as a threatened species in Oman’s Plant Red Data Book (Patzelt, 2015a). More recently, wild olive has been assessed as Vulnerable (VU) in the United Arab Emirates National Red List of Vascular Plants (Allen et al., 2021).

Wild olive typically inhabits isolated mountainous environments that are fragile and highly susceptible to various threats (Al-Charaabi et al., 2013; Patzelt, 2015b; Patzelt, 2015c). Observations in Oman indicate that anthropogenic pressures such as urban expansion, overgrazing, illegal wood harvesting, and the introduction of non-native species, along with natural stressors like pests, diseases, and climate change, have collectively contributed to habitat degradation (Al-Jabri et al., 2024). These factors are likely to accelerate genetic erosion by reducing population sizes and gene flow, as observed in *O. europaea* subsp. *laperrinei* populations of the Saharan mountains where natural regeneration has ceased (Baali-Cherif and Besnard, 2005; Anthelme et al., 2008).

Despite its ecological and cultural significance, information on the genetic diversity of wild olive in Oman remains limited. This knowledge gap restricts the understanding of its adaptive traits and hinders the development of effective conservation measures. Previous research on Omani wild olives has been geographically restricted to the Western Hajar Mountains (Habib et al., 2021), where substantial genetic variation was observed among populations. That study reported no clear evidence of population substructure, suggesting that pollen- and seed-mediated gene flow may have historically facilitated connectivity across the region.

Wild olive (*Olea europaea* subsp. *cuspidata*) plays a crucial ecological role in Omani mountain ecosystems, serving as a food source for wildlife and providing traditional medicinal and timber resources for local communities. Understanding its genetic diversity is therefore essential to evaluate its adaptive capacity and resilience to ongoing environmental change. The present study investigated the genetic diversity and population structure of wild olive populations across different regions of Oman using single nucleotide polymorphism (SNP) markers to provide a comprehensive assessment of natural genetic variation in this subspecies in support of the development of national conservation and management strategies.

## Methods

### Plant material

**Figure 1:**
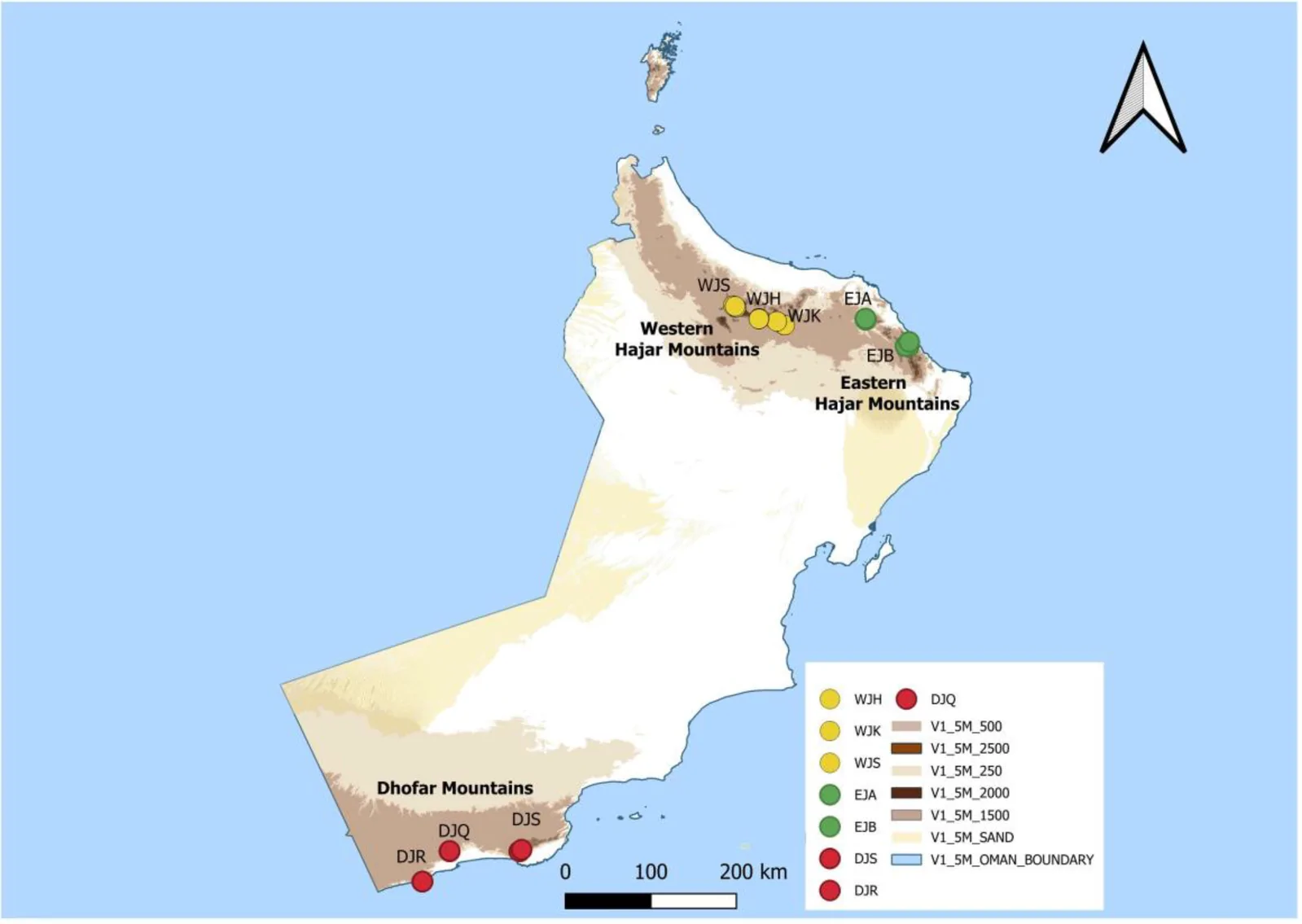
Geographic distribution of sampled wild olive (*Olea europaea* subsp. *cuspidata*) populations in Oman. Sampling was conducted across three major mountain ranges: the Western Hajar Mountains (WJH, WJK, WJS; yellow circles), the Eastern Hajar Mountains (EJA, EJB; green circles), and the Dhofar Mountains (DJQ, DJR, DJS; red circles). Elevation is indicated by shading, and the Oman national boundary is highlighted in light blue.

A total of 48 wild olive (*Olea europaea* subsp. *cuspidata*) leaf samples were collected from eight native populations distributed across three major mountain ranges in Oman -the Western Hajar Mountains, the Eastern Hajar Mountains, and the Dhofar Mountains- between June and August 2020, following standardized field sampling procedures. These eight populations were strategically chosen to cover the full latitudinal and altitudinal range of the species in Oman, ensuring representation of both major evolutionary lineages (North and South). Although the total number of individuals per population was limited due to logistical constraints, the genome-wide SNP dataset provided sufficient statistical power to accurately estimate genetic diversity and population structure, as demonstrated in previous studies where large SNP panels compensate for smaller sample sizes (Willing et al., 2012). A map showing the geographic locations of all eight sampled populations across Oman’s mountain ranges is provided in **Figure 1** to illustrate their distribution and spatial coverage. Within the Eastern Hajar Mountains, the Jabal Abyad (EJA) site included the Wadi Sareen Nature Reserve; accordingly, collections at this locality were conducted under a permit issued by the Office for Conservation of the Environment (Permit No. 17/SN 2020). All other sampling locations were outside designated protected areas and therefore did not require specific collection permits. Herbarium voucher specimens for all populations were collected and deposited at the University of Reading Herbarium (RNG), United Kingdom.

Leaf samples were collected from three populations in each of the Dhofar Mountains (Jabal Qara, Jabal Qamar, and Jabal Samhan) and the Western Hajar Mountains (Jabal Akhdar, Jabal Shams, and Jabal Hatt), and from two populations in the Eastern Hajar Mountains (Jabal Abyad and Jabal Bani Jabir).

All collected samples consisted of fresh, healthy leaves which were immediately dried with silica gel in the field to preserve DNA integrity until extraction.

### DNA extraction

Total genomic DNA was extracted from all 48 samples. Dried leaf material (20–50 mg) was ground using a TissueLyser II (QIAGEN, Manchester, UK) for two cycles at 30 Hz for one minute each. DNA extraction followed the cetyltrimethylammonium bromide (CTAB) protocol of Doyle and Doyle (1987), with modifications based on Tel-Zur et al (Tel-Zur et al., 1999). and further optimized according to Yooprasert (2021) to improve DNA yield and purity. Specifically, three rinses in ice-cold sorbitol buffer (100 mM Tris-HCl pH 8, 5 mM EDTA pH 8, 0.35 M sorbitol) were performed prior to incubation in CTAB buffer, followed by the addition of 3 M potassium acetate (pH 5.5) before the chloroform–isoamyl alcohol extraction step. DNA quality and fragment integrity were assessed using 0.7% agarose gel electrophoresis stained with GelRed^®^ (Biotium, Fremont, USA) and compared against HyperLadder™ 1 kb (Bioline Reagents Ltd., London, UK) using either Tris-Borate-EDTA (TBE) or Sodium Borate (SB) buffers. DNA concentration and purity were quantified using a NanoDrop™ Lite spectrophotometer (Fisher Scientific UK Ltd., Loughborough, UK) and a Qubit^®^ fluorometer (Thermo Fisher Scientific, Waltham, Massachusetts, USA).

### DNA library construction and sequencing

The DNA libraries were pooled and enriched using the Angiosperms353 target capture kit (myBaits^®^ Target Sequence Capture Kit; Daicel Arbor Biosciences, Michigan, USA) following the manufacturer’s protocol (version 4.0; http://www.arborbiosci.com/mybaits-manual). SNP markers were selected because they are bi-allelic, abundant throughout the genome, and provide high resolution for detecting genetic variation. Additionally, SNP genotyping can be easily automated, allowing for efficient and reproducible large-scale analysis (Park et al., 2022).

### Data processing

Raw Illumina NovaSeq S4 paired-end (150 bp) reads were quality filtered and trimmed using Trimmomatic v0.39 (Bolger et al., 2014) by removing low-quality bases (Phred score< 28), discarding reads shorter than 50 bp, and eliminating sequencing adapters. The Angiosperms353 target genes were recovered from the trimmed reads using HybPiper v1.3 (Johnson and etal, 2016) and the ‘mega353’ target nucleotide file (McLay and etal, 2021). Briefly, HybPiper identifies potential matches between trimmed reads and target genes using BLASTX (Camacho et al., 2009), which allows for substantial phylogenetic divergence between the reference targets and sample reads. The program then sorts reads by gene and performs *de novo* assembly for each locus individually. Two sequence types were generated by HybPiper: coding sequences (CDS) corresponding to the target genes, and ‘*supercontigs*’ containing both exon sequences and flanking non-coding regions (i.e. introns and untranslated sequences). The final reference set was generated by selecting, for each gene, the longest *supercontig* recovered among all samples. The sample from Jabal Abyad (EA4) yielded the longest and most complete *supercontigs*, which were used as the reference for downstream analyses.

### Variant detection and filtering

To characterize genomic variation across different genome compartments, we generated two distinct SNP datasets: a nuclear dataset and a plastid dataset. Genetic variants among wild olive samples were identified using the Genome Analysis Toolkit (GATK v4) (McKenna et al., 2010). For the nuclear SNP dataset, the nuclear reference set consisting of the longest Angiosperms353 supercontigs recovered from sample EA4 was used to detect genomic variation following the variantcall.sh pipeline (Slimp et al., 2021). Simultaneously, to identify plastid single nucleotide polymorphisms (cpSNPs) for haplotype and network analyses, the chloroplast genome of *Olea europaea* cultivar Bianchera (NC_013707) was employed as a separate reference to capture “off-target” plastid reads.

The *variantcall.sh* pipeline combines Samtools and GATK to align paired-end reads to their respective reference sequences and call genotypes for each individual. The output comprised variant call format (VCF) files containing both single nucleotide polymorphisms (SNPs) and insertions/deletions (indels). Subsequently, individual genomic VCF (GVCF) files were merged into a single cohort VCF using the *GenotypesToPCA.sh* script (Johnson et al., 2021).

**Figure 2:**
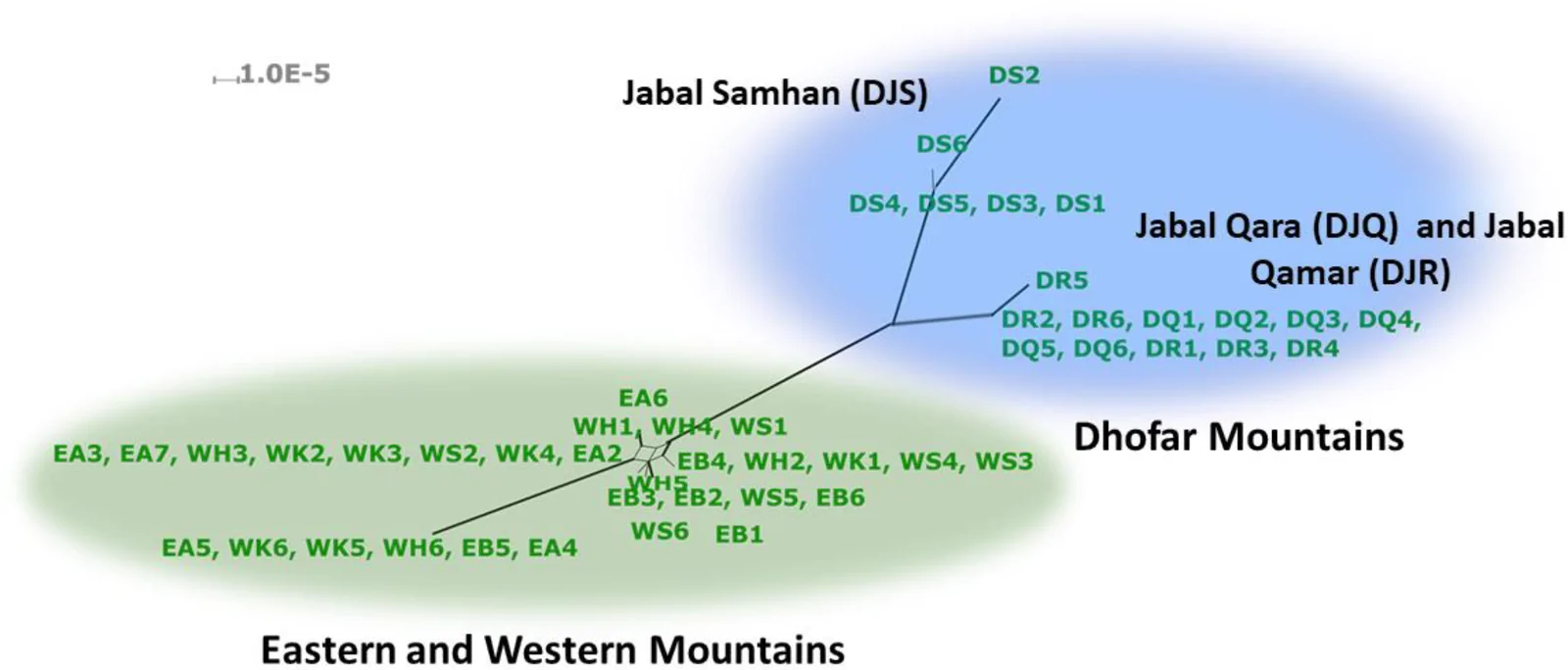
NeighborNet network generated using complete plastid sequences of 48 wild olive accessions from three mountain ranges (Eastern Hajar, Western Hajar and Dhofar) using SplitsTree version 4.17.0. Blue color indicates wild olive accessions from Dhofar mountains (Jabal Qara (DJQ), Jabal Qamar (DJR) and Jabal Samhan (DJS), green color shows the wild olive accessions from Western Hajar mountains (Jabal Hatt (WJH), Jabal Akhdar (WJK) and Jabal Shams (WJS) and Eastern Hajar mountains (Jabal Abyad (EJA) and Jabal Bani Jabir (EJB).

For the nuclear dataset, hard filtering was applied to ensure high-confidence variant calls by excluding indels and removing low-quality SNPs that did not meet the following thresholds: QualByDepth (QD)< 5.0, FisherStrand (FS) > 60.0, RMSMappingQuality (MQ)< 40.0, MappingQualityRankSum (MQRankSum)< -12.5, and ReadPosRankSum< -8.0 (Johnson et al., 2021). The resulting filtered nuclear dataset was used for downstream population structure and diversity analyses, while the cpSNP dataset was specifically utilized for the haplotype network construction (**Figure 2**).

**Figure 3:**
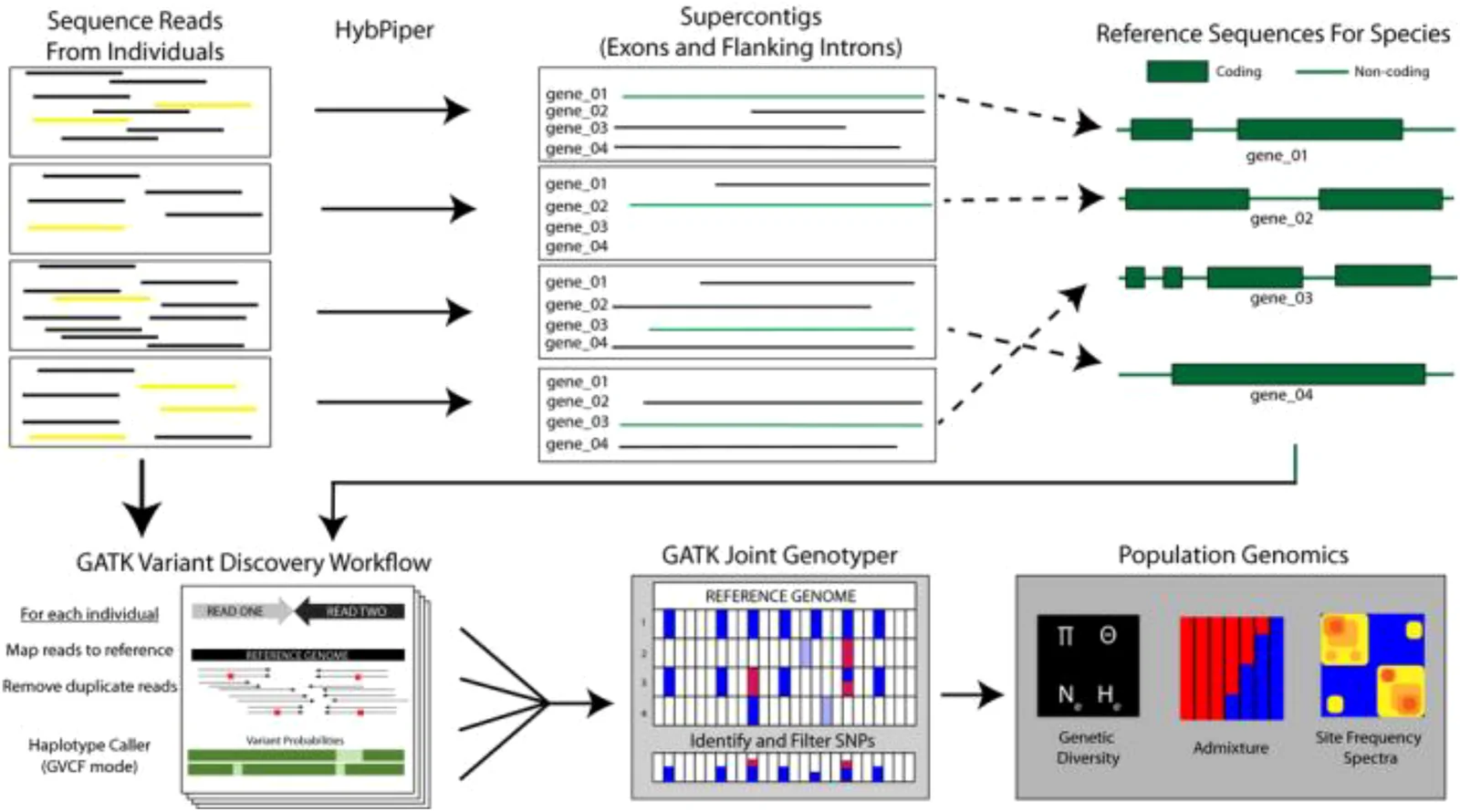
Workflow for detecting SNPs from target capture data. Reference sequences were constructed by selecting the longest supercontig from each gene for each individual. The workflow includes read mapping, variant calling, and filtering steps implemented using HybPiper and GATK pipelines. *Adapted from Slimp* et al (Slimp et al., 2021).

The general workflow for SNP detection from target capture data is illustrated in **Figure 3**. Reference sequences were generated by selecting the longest supercontig from each gene for each individual. The process included read mapping, variant calling, and filtering steps as implemented through HybPiper and GATK pipelines, adapted from Slimp et al (Slimp et al., 2021).

### Population genetic and bioinformatics analysis

To assess the genetic health and connectivity of the eight *Olea europaea* populations, a suite of population genetic indices was calculated. The bioinformatics analysis was performed in R v4.3.2. The raw VCF file was processed and converted into a genomic data frame using the *vcfR* and *adegenet* packages.

Population genetic structure was evaluated using 56,410 high-quality SNPs. These were retained following a rigorous two-stage filtering process: first, GATK hard-filtering was applied to ensure high-confidence variant calls, followed by filtering for a Minor Allele Frequency (MAF) of 0.001 and a maximum missing data threshold of 30% per locus. To preserve the maximum phylogenetic signal and rare allelic variants critical for resolving the deep evolutionary divergence between the Eastern Hajar, Western Hajar, and Dhofar lineages, Linkage Disequilibrium (LD) pruning was not implemented, following the recommendations of Linck and Battey (2019). Within-population diversity was quantified by calculating the number of polymorphic sites, expected heterozygosity (Hs), and the inbreeding coefficient (F_IS_) for each Jabal using the *basic.stats* function in the *hierfstat* package. Nucleotide diversity (pi) was derived from the mean expected heterozygosity across all loci, serving as a robust proxy for diversity in this high-density SNP dataset.

To evaluate regional differentiation, the Global Fixation Index (F_ST_) was quantified using the Weir and Cockerham (1984) estimator to account for unequal sample sizes. Hierarchical Analysis of Molecular Variance (AMOVA) was performed at two scales: among mountain ranges (**Table 1**) and among specific geographical regions (Dhofar Mountains, Eastern Hajar Mountains, and Western Hajar Mountains; **Table 2**) to reconcile global F_ST_ values with localized variance partitioning. Finally, the rate of Gene Migration (Nm) was estimated using the formula:

**Table 1:** Analysis of Molecular Variance (AMOVA) results for genetic variation among and within eight wild olive populations across Oman.

Source of variation	df	Sum of squares	Mean square	Sigma	Percentage variation	Phi	P value
Among populations	7	152,641.0	21,805.86	745.93	28.14	0.2814	0.001
Within populations	40	451,690.2	11,292.26	1,903.05	71.86		
Total	47	604,331.2	12,858.11	2,648.98	100		

Dhofar Mountains: DJQ, Jabal Qara; DJR, Jabal Qamar; DJS, Jabal Samhan; Eastern Hajar Mountains: EJA, Jabal Abyad; EJB, Jabal Bani Jabir; Western Hajar Mountains: WJH, Jabal Hatt; WJK, Jabal Akhdar; WJS, Jabal Shams. Sigma represents variance components, and *Phi* denotes population differentiation statistics.

**Table 2:** Analysis of Molecular Variance (AMOVA) for genetic variation among and within three mountain ranges: Dhofar, Eastern Hajar, and Western Hajar Mountains.

Source of variation	df	Sum of squares	Mean square	Sigma	Percentage variation	Phi	P value
Among mountains	2	275897.2	137948.588	8288.003	52.78798	0.5278798	0.001
Within mountains	45	333564.6	7412.546	7412.546	47.21202		
Total	47	609461.8	12967.271	15700.549	100.00000		

Sigma represents variance components, and *Phi* denotes population differentiation statistics.


$$Nm=\frac{1-FST}{4FST}$$

This calculation was used to determine whether contemporary gene flow is sufficient to counteract the effects of genetic drift (Nm > 1) or if the populations are evolving in isolation (Nm< 1).

### Population structure analysis and genetic diversity

The nuclear SNP VCF file was converted to STRUCTURE format using PGDSpider version 2.1.1.5 (Lischer and Excoffier, 2012). Population structure was inferred using STRUCTURE version 2.3.4 (Pritchard et al., 2000) under the admixture model. Analyses were conducted for K values ranging from 1 to 8, with five independent replicates for each K. Each run used a burn-in period of 100,000 generations followed by 100,000 Markov Chain Monte Carlo (MCMC) iterations.

Given the relatively large dataset and high number of SNP markers, computational efficiency was optimized by limiting the burn-in and iteration numbers to 10,000 and 50,000, respectively (Wang et al., 2013). The optimal K value was determined using the *ad hoc delta K* (ΔK) method (Evanno et al., 2005) implemented in the R package POPHELPER (version 2.3.1 (Francis, 2017);), which was also used to visualize STRUCTURE results.

Discriminant Analysis of Principal Components (DAPC) and Principal Component Analysis (PCA) were used to estimate the population structure of wild olives across Oman using the R package *adegenet* version 2.1.10 (Jombart, 2008). To visualize individual assignments, a composite stacked bar plot illustrating the probability of population membership on the Y-axis was generated.

The Analysis of Molecular Variance (AMOVA) and hierarchical partitioning of genetic variance were implemented using the R package *poppr* (Kamvar et al., 2014), with data integration handled via *vcfR* (Knaus and Grünwald, 2017). Genetic differentiation (F_ST_) between the eight Omani populations was quantified using the Weir and Cockerham unbiased estimator within the *StAMPP* package (Weir and Cockerham, 1984), which is specifically optimized for high-density SNP datasets. Estimates of within-population genetic variation, including expected heterozygosity (Hs) and the inbreeding coefficient (F_IS_), were calculated using the *hierfstat* package (Purcell et al., 2007) to ensure statistical consistency across all genomic indices.

In addition, the correlation between genetic and geographic distances among the 48 Omani samples was assessed using a Mantel test (Smouse et al., 1986) with 100,000 permutations, based on chloroplast SNPs (cpDNA), as implemented in the R package *vegan* (Oksanen et al., 2018).

### Phylogenetic analysis

The filtered nuclear SNPs (nrDNA) VCF file for the Omani wild olive dataset was converted to a NEXUS format using the *vcf2phylip.py* script (Ortiz, 2019). An unrooted splitstree was then constructed using the Neighbor-Net method implemented in *SplitsTree* version 4.17.0 (Huson and Bryant, 2006).

### Haplotype network analysis

The filtered plastid SNPs (cpDNA) VCF file for the Omani wild olive dataset was converted to a FASTA format using the *vcf2phylip.py* script (Ortiz, 2019). Relationships among haplotypes were inferred using the Templeton, Crandall, and Sing (TCS) network method (Templeton et al., 1992) implemented in Population Analysis with Reticulate Trees (*PopART* (Leigh et al., 2015); https://popart.maths.otago.ac.nz/; accessed 30 May 2022). Geographic coordinates of samples were used to visualize the spatial distribution of genetic clusters and haplotypes.

## Results

A total of 61,723 biallelic SNP markers were initially identified across the eight wild olive populations analyzed. After stringent hard filtering, 56,410 high-quality biallelic SNPs were retained for selected downstream analyses.

### Population structure and genetic diversity analyses

**Figure 4:**
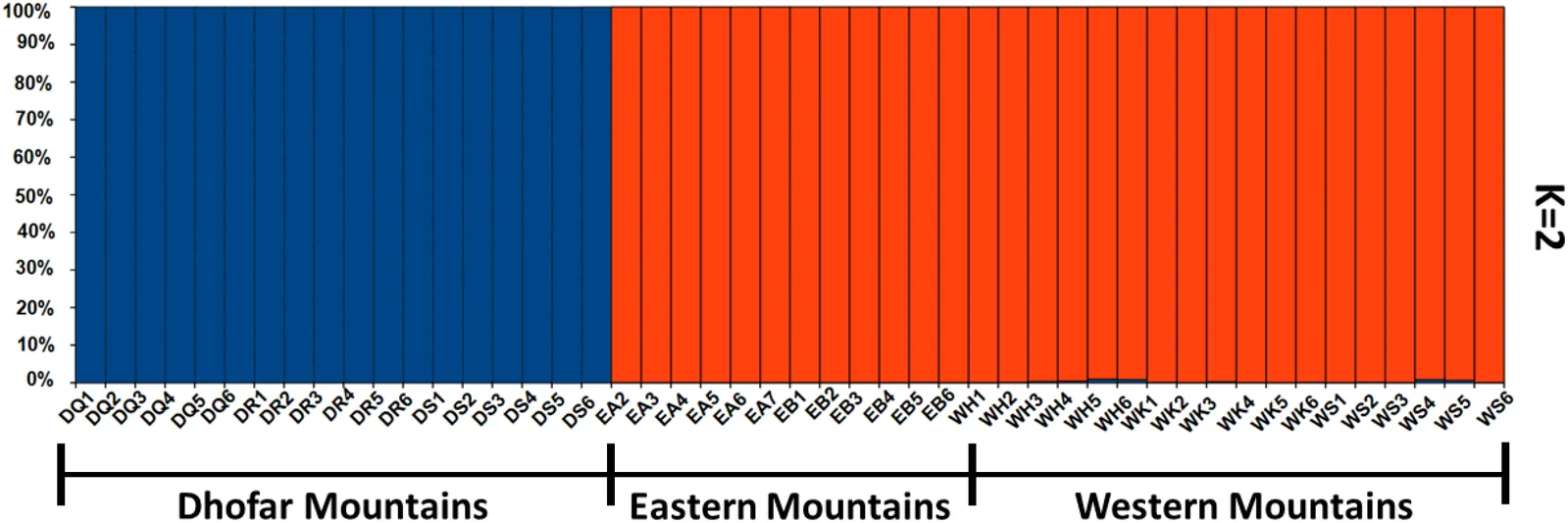
STRUCTURE bar plot of population assignment for 48 wild olive accessions from eight populations across Oman at *K* = 2, based on 61,723 SNPs using the longest supercontig of EA4 as the reference. Accessions represented in blue correspond to Cluster 1 (Dhofar Mountains), whereas those in red correspond to Cluster 2 (Eastern and Western Hajar Mountains). The y-axis represents the proportion of membership of each genotype to the inferred genetic clusters.

The optimal ΔK value was detected at *K* = 2 (**Figure 4**), indicating the presence of two distinct genetic clusters among wild olive populations. The first cluster comprised accessions from the Dhofar Mountains (blue), whereas the second cluster included accessions from the Western and Eastern Hajar Mountains (red). The expected heterozygosity was slightly lower in Cluster 1 (*He* = 0.18) compared to Cluster 2 (*He* = 0.21). In contrast, genetic differentiation among populations was higher in Cluster 1 (F_ST_= 0.49) than in Cluster 2 (F_ST_= 0.34) (**Table 3**).

**Table 3:** Results of STRUCTURE analysis for 48 *Olea europaea* subsp. *cuspidata* accessions, showing the fixation index (F_ST_), expected heterozygosity (He), and the number of genotypes assigned to each inferred cluster.

Population	Inferred clusters	Mean fixation index (FST)	Expected heterozygosity (He)	No. of genotypes
Cluster 1 (Dhofar)	0.376	0.489	0.181	18
Cluster 2 (Eastern and Western Hajar mountains)	0.624	0.339	0.210	30

**Figure 5:**
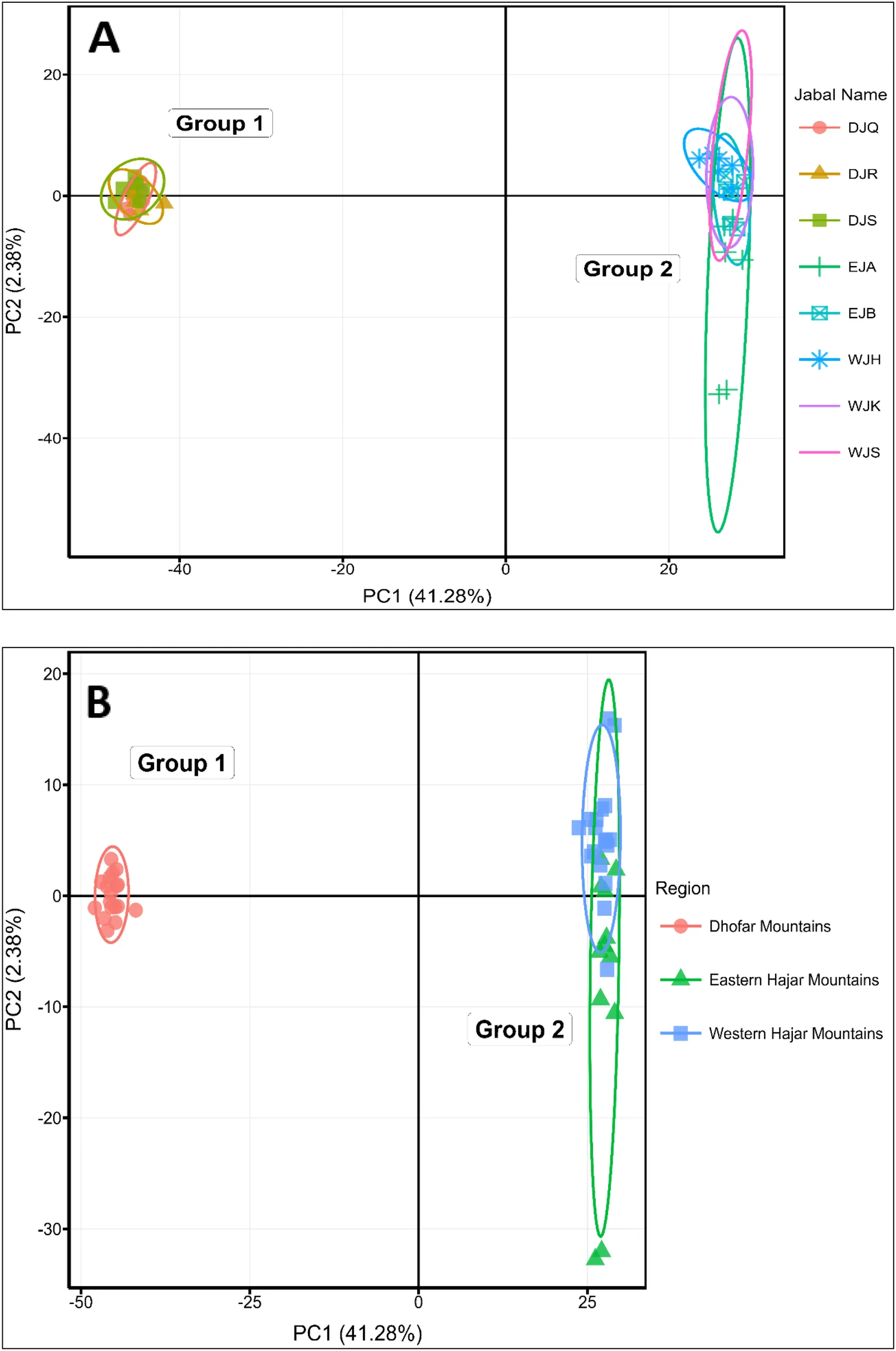
Principal Component Analysis (PCA) of 48 wild olive (*Olea europaea* subsp. *cuspidata*) accessions from eight populations across Oman based on 61,723 SNPs. **(A)** Clustering by population: DJQ, Jabal Qara; DJR, Jabal Qamar; DJS, Jabal Samhan; EJA, Jabal Abyad; EJB, Jabal Bani Jabir; WJH, Jabal Hatt; WJK, Jabal Akhdar; and WJS, Jabal Shams. **(B)** Clustering by mountain ranges: Dhofar Mountains; and the Eastern and Western Hajar Mountains.

The Principal Component Analysis (PCA) revealed that the Western and Eastern Hajar Mountain populations clustered closely together, whereas the Dhofar Mountain populations were clearly distinct from both Hajar populations (**Figures 5A, B**). Samples from Jabal Abyad in the Eastern Hajar Mountains exhibited greater variability than those from any other location in Oman. Overall, the PCA results were consistent with the STRUCTURE analysis, supporting the presence of two major genetic clusters across Oman.

**Figure 6:**
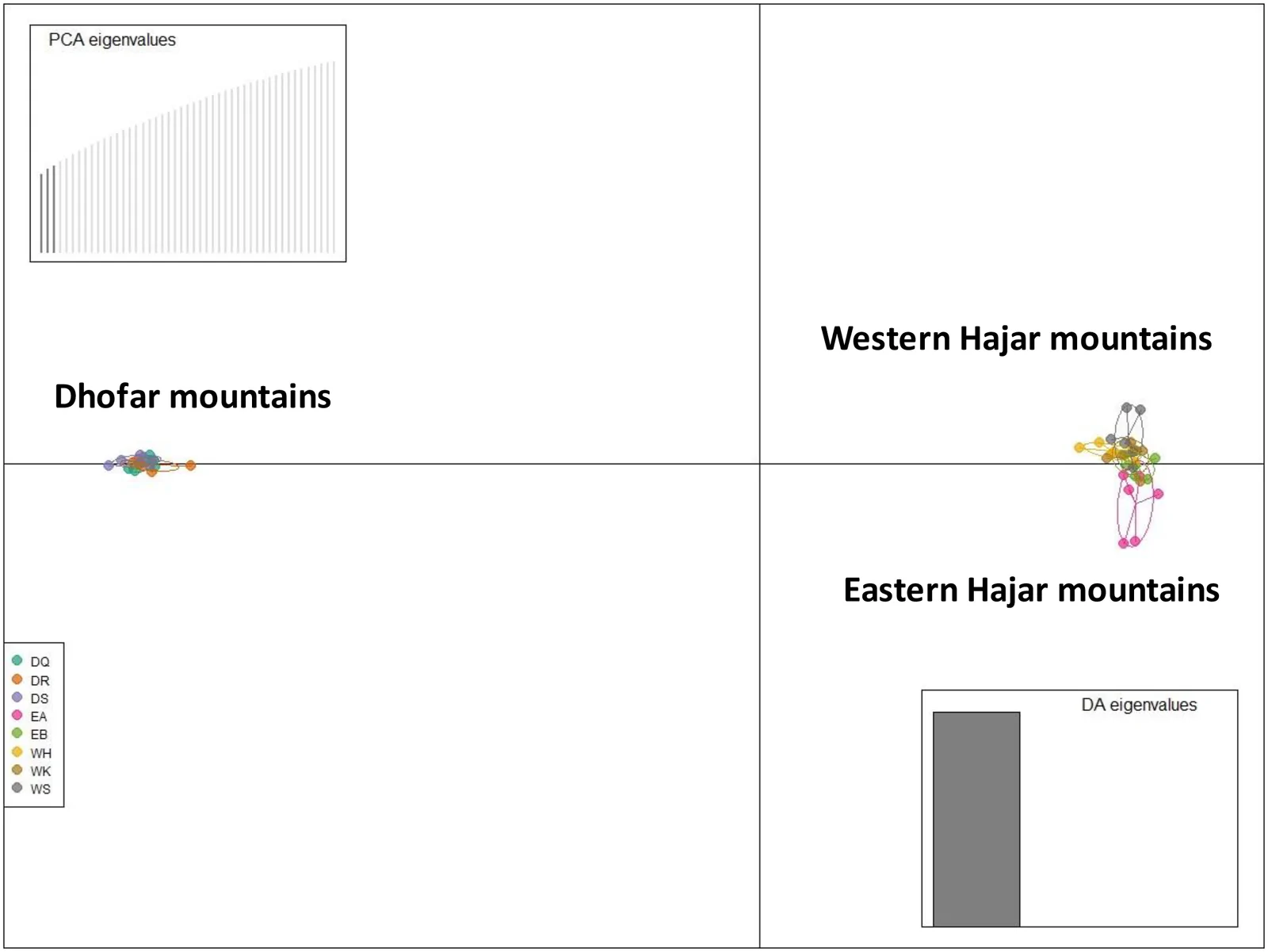
Discriminant Analysis of Principal Components (DAPC) of 48 wild olive (*Olea europaea* subsp. *cuspidata*) accessions from Oman based on 61,723 SNPs derived from variant calling using the longest supercontig (EA4) as the reference. Ellipses represent the eight sampled populations (one per location; see key), and each point corresponds to a single accession.

**Figure 7:**
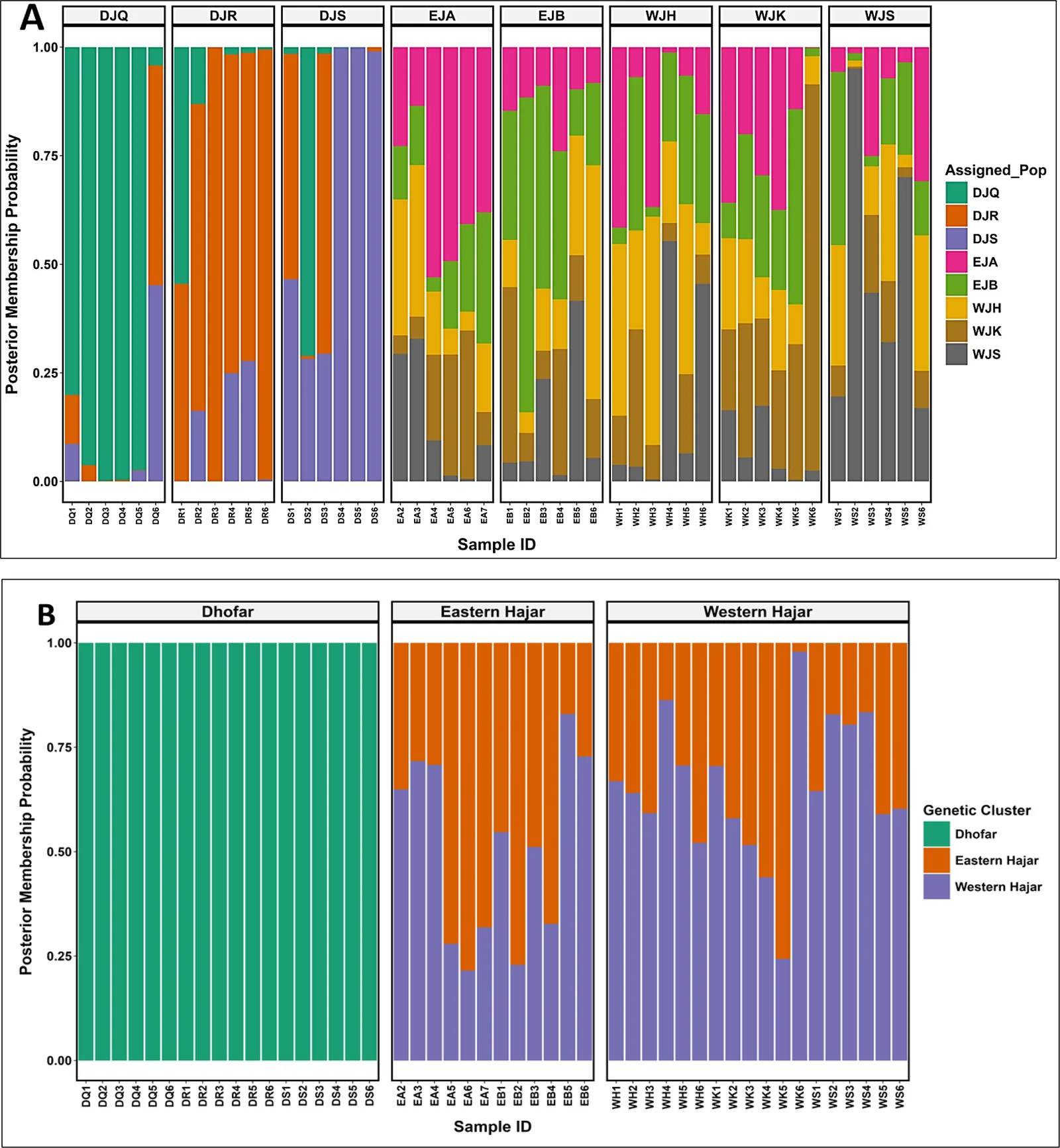
Composite DAPC plots of 48 wild olive (*Olea europaea* subsp. *cuspidata*) accessions from Oman based on SNPs derived from the longest supercontig reference (EA4), showing mixed ancestry between the Eastern and Western Hajar Mountains. **(A)** Composite plot of wild olive accessions from the eight sampled populations across Oman (DJQ, DJR, DJS, WJH, WJK, WJS, EJA, and EJB). **(B)** Composite plot showing population clustering among the three major mountain ranges (Western Hajar, Eastern Hajar, and Dhofar Mountains).

Discriminant Analysis of Principal Components (DAPC) grouped the wild olive accessions into two distinct clusters (**Figure 6**). Samples from the Eastern and Western Hajar Mountains were positioned on the right side of the DAPC axis, whereas the Dhofar samples were placed on the left side. Considerable overlap was observed between the Eastern and Western Hajar Mountain accessions, while the Dhofar populations were clearly separated from both Hajar groups. The population membership assignment inferred from the DAPC composite plot (**Figure 7**) supported the STRUCTURE and PCA results. All populations from the Western Hajar Mountains exhibited admixture with those from the Eastern Hajar Mountains (**Figure 7A**). Admixture was also detected within the Dhofar Mountain populations; however, no admixture was observed between Dhofar and the Eastern or Western Hajar populations (**Figure 7B**).

Analysis of 56,410 high-quality nuclear SNPs across eight populations of *Olea europaea* subsp. *cuspidata* in Oman revealed a robust genetic structure with high genomic resolution. Within-population diversity was consistent across the mountain ranges, with Expected Heterozygosity (Hs) and Nucleotide Diversity (pi) ranging from 0.0865 to 0.1082, and the number of polymorphic sites per population exceeding 9,800. Notably, all populations exhibited negative Inbreeding Coefficients (F_IS_), ranging from -0.1543 to -0.3104, indicating a significant excess of heterozygosity consistent with an outcrossing mating system.

Despite this internal genetic health, a high Global Fixation Index (F_ST_ = 0.3093) and a low Gene Migration rate (Nm = 0.5584) demonstrate restricted gene flow between the Northern Hajar and Southern Dhofar lineages. This regional isolation was further substantiated by the Hierarchical Analysis of Molecular Variance (AMOVA), which revealed that 28.14% of the total genetic variance is partitioned among mountain ranges (Phi = 0.2814, P = 0.001; **Table 1**). Even when evaluated at a finer regional scale (Dhofar, Eastern Hajar, and Western Hajar), the genetic structure remained highly significant, confirming that the central desert acts as a formidable barrier to gene flow (**Table 2**).

These results confirm that while Omani wild olives are not currently suffering from inbreeding, the Southern populations (DJQ, DJR, and DJS) represent a genetically isolated and unique Evolutionarily Significant Unit (ESU) that must be managed independently to preserve the species’ full evolutionary potential in the region (**Table 4**).

**Table 4:** Mean genetic diversity indices for wild olive (*Olea europaea* subsp. *cuspidata*) accessions from eight populations across Oman based on nuclear SNPs.

Genetic index	DJQ	DJR	DJS	EJA	EJB	WJH	WJK	WJS	Overall
No. of Polymorphic Sites	9,814	9,935	10,166	11,572	14,767	14,812	14,275	13,764	56,410
Genetic Diversity (Hs)	0.0865	0.0895	0.0881	0.0925	0.1055	0.1082	0.1031	0.1020	0.0970
Inbreeding Coeff. (F_IS_)	-0.2922	-0.2718	-0.3104	-0.1698	-0.1543	-0.1662	-0.1564	-0.1554	-0.2100
Nucleotide Diversity (Pi)	0.0865	0.0895	0.0881	0.0925	0.1055	0.1082	0.1031	0.1020	0.0970
Fixation Index (F_ST_)	—	—	—	—	—	—	—	—	0.3093
Gene Migration (Nm)	—	—	—	—	—	—	—	—	0.5584

**Figure 8:**
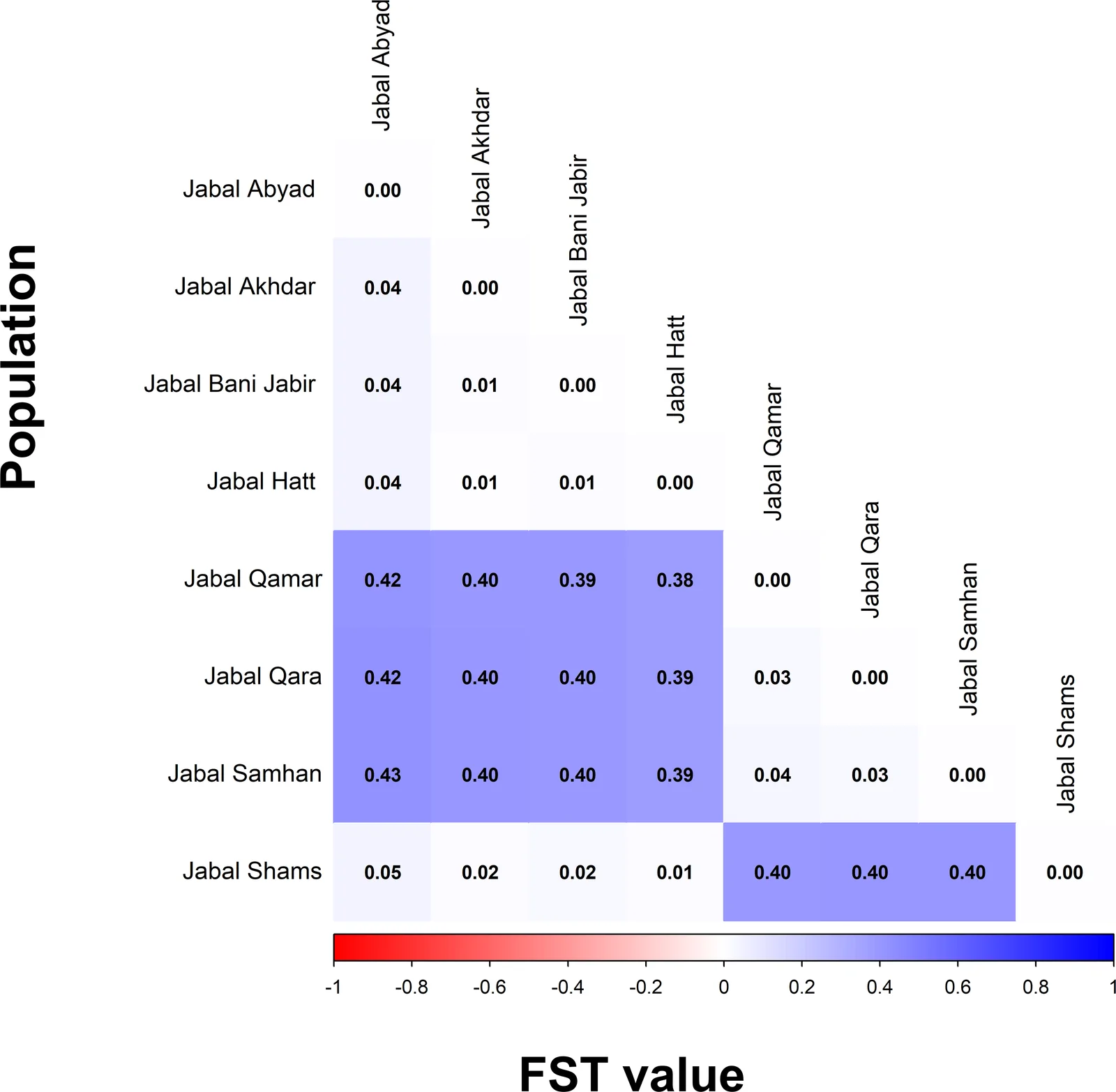
Heatmap of pairwise F_ST_ values (Weir and Cockerham, 1984) among eight populations of wild olive (*Olea europaea* subsp. *cuspidata*) in Oman, calculated from 56,410 nuclear SNPs. The color scale represents the degree of genetic differentiation, ranging from low (red; F_ST_< 0.05) to high (blue; F_ST_ > 0.40). Numbers within the cells indicate the specific pairwise F_ST_ estimates. A prominent genetic break is visible between the southern Dhofar Mountains (DJQ, DJR, DJS) and the northern Hajar Mountains (EJA, EJB, WJH, WJK, WJS), while populations within the Hajar range exhibit high genetic connectivity and low differentiation.

The pairwise F_ST_ values ranged from near zero (e.g., 0.00 to 0.05 among populations within the Hajar Mountains) to higher values between populations from Dhofar and the Hajar Mountains (0.38 to 0.43), indicating strong genetic differentiation (**Figure 8**). The highest genetic differentiation was observed between the southern Dhofar populations (DJQ, DJR, DJS) and both Eastern and Western Hajar populations, suggesting that the Dhofar populations are genetically isolated and may represent a distinct lineage or subspecies. Conversely, the lowest pairwise F_ST_ values occurred among populations within the Eastern and Western Hajar Mountains, reflecting ongoing gene flow and closer genetic connectivity in these regions.

The Analysis of Molecular Variance (AMOVA; **Table 1**) confirmed significant genetic differentiation among the eight populations (ΦST = 0.2814, P = 0.001), with the majority of genetic variation found within populations (71.86%) compared to (28.14%) among populations. This pattern supports the presence of population structure while indicating substantial genetic diversity is maintained within populations.

### Correlation between genetic and geographic distances (Mantel test)

**Figure 9:**
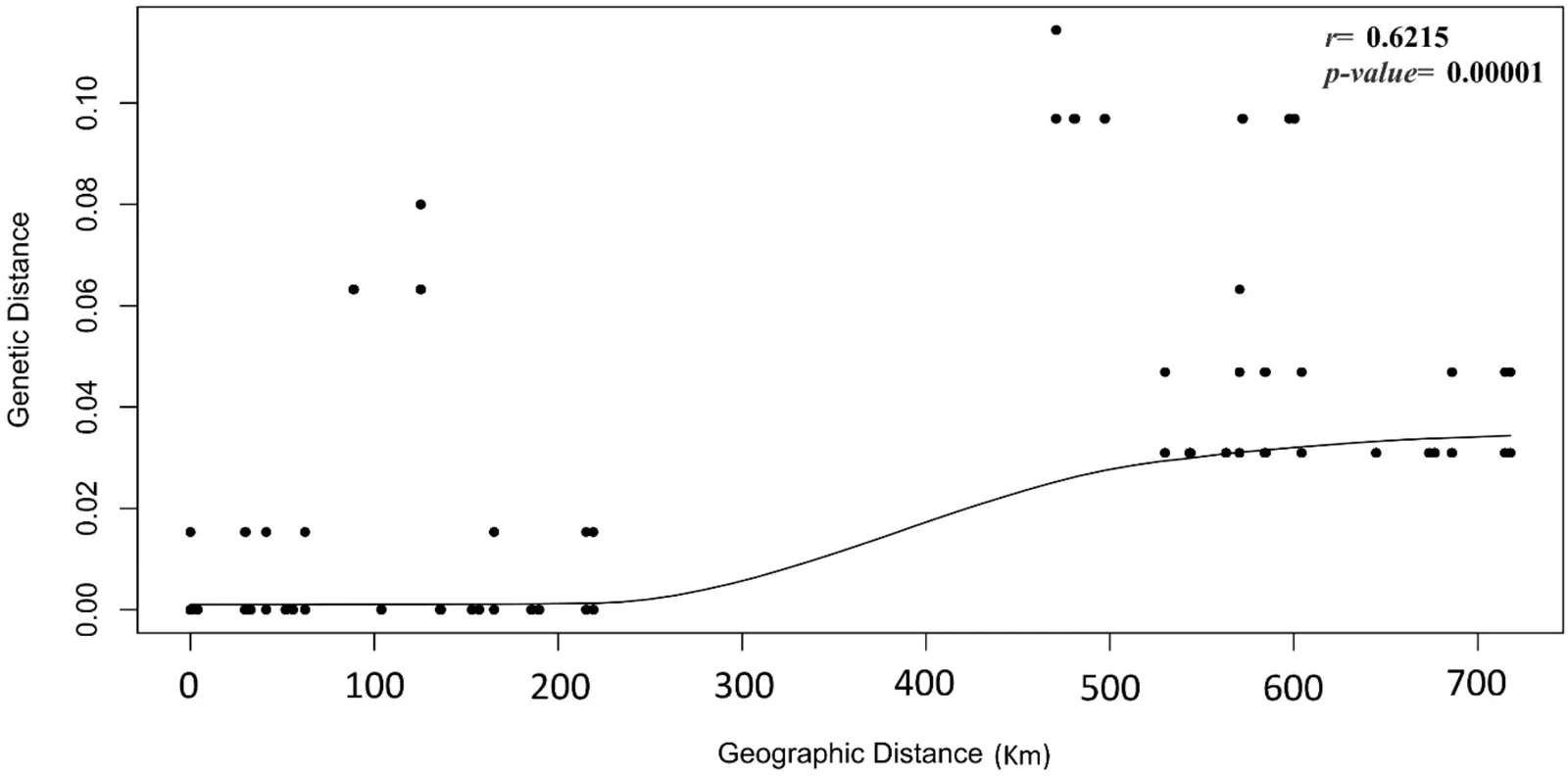
Mantel test showing the correlation between geographic and genetic distances among 48 wild olive (*Olea europaea* subsp. *cuspidata*) accessions across Oman based on chloroplast SNPs (*r* = 0.6215, *P* = 0.00001).

The Mantel test based on complete chloroplast SNPs revealed a strong and significant positive correlation between genetic distance (F_ST_/(1 – F_ST_)) and geographic distance among wild olive populations across Oman (*r* = 0.6215, *P* = 0.00001; **Figure 9**). This indicates a clear isolation-by-distance pattern within the Omani populations.

### Phylogenetic analysis

**Figure 10:**
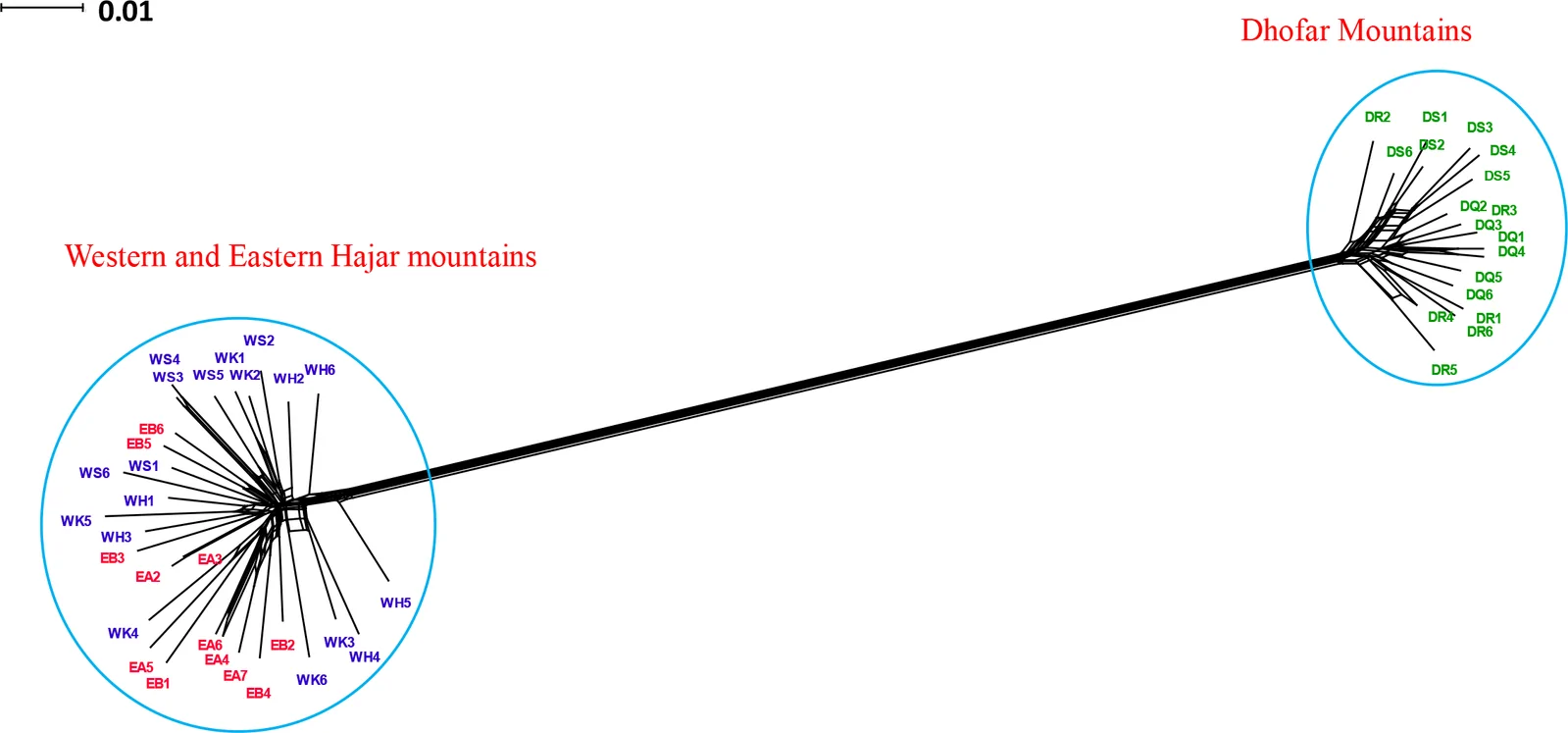
NeighborNet network generated using 61,723 nuclear SNPs of wild olive across Oman using *SplitsTree* version 4.17.0.

The NeighborNet network analysis grouped the wild olive accessions from Oman into two distinct clusters. Cluster I comprised accessions from the Eastern and Western Hajar Mountains, while Cluster II consisted of accessions from the Dhofar Mountains (**Figure 10**). This clear separation indicates a strong genetic differentiation between the Hajar and Dhofar mountain populations, supporting the results obtained from the STRUCTURE, PCA, and DAPC analyses.

### Haplotype analysis

**Figure 11:**
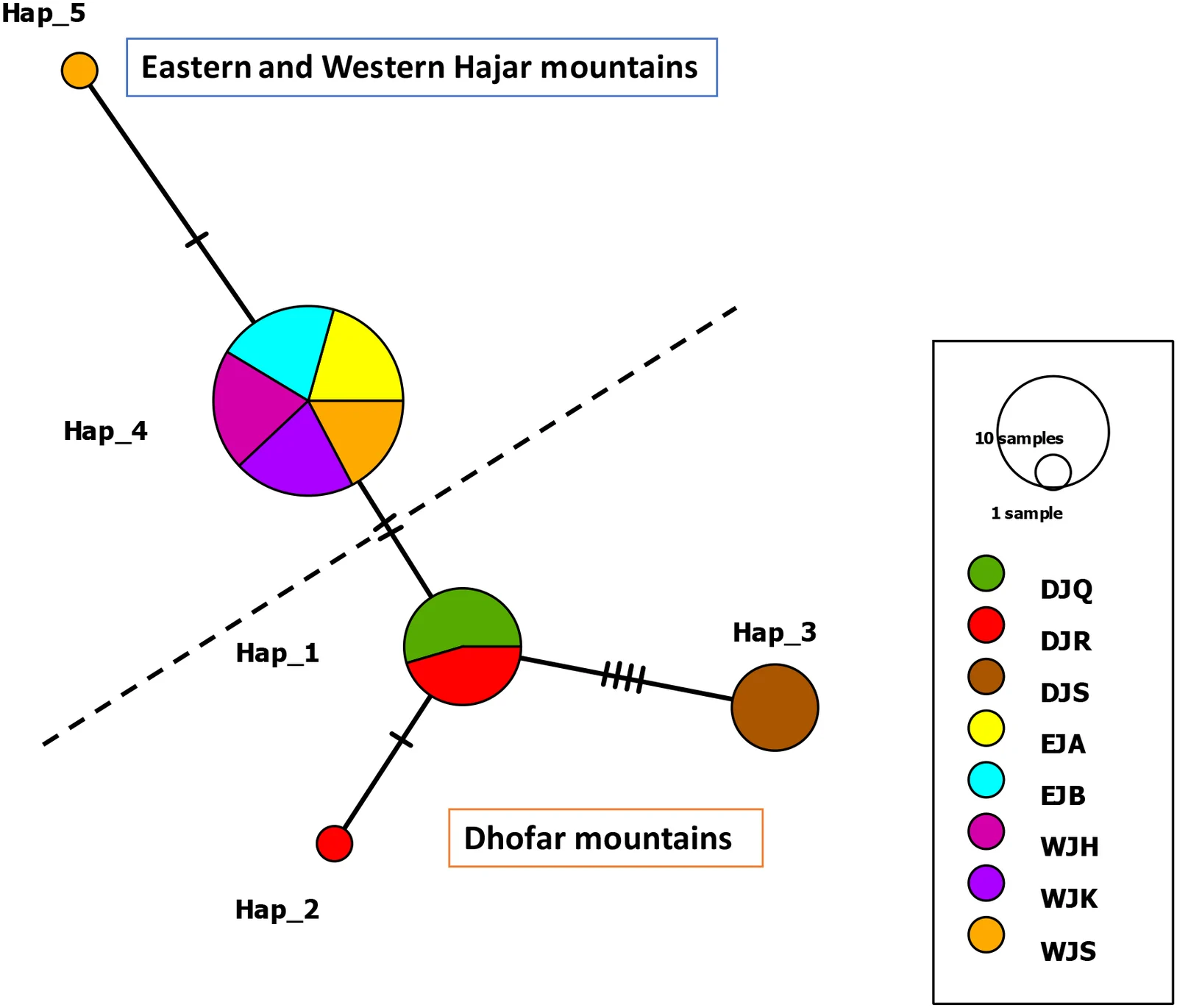
TCS haplotype network of the complete chloroplast genome sequences (cpDNA) for wild olive across Oman based on chloroplast SNPs. The size of each circle represents the number of individuals sharing that haplotype, and crosshatches on the connecting lines indicate the number of nucleotide differences between haplotypes. Colour codes denote the eight wild olive populations across Oman: DJQ (Jabal Qara), DJR (Jabal Qamar), DJS (Jabal Samhan) from the Dhofar Mountains; EJA (Jabal Abyad) and EJB (Jabal Bani Jabir) from the Eastern Hajar Mountains; and WJH (Jabal Hatt), WJK (Jabal Akhdar), and WJS (Jabal Shams) from the Western Hajar Mountains.

**Figure 12:**
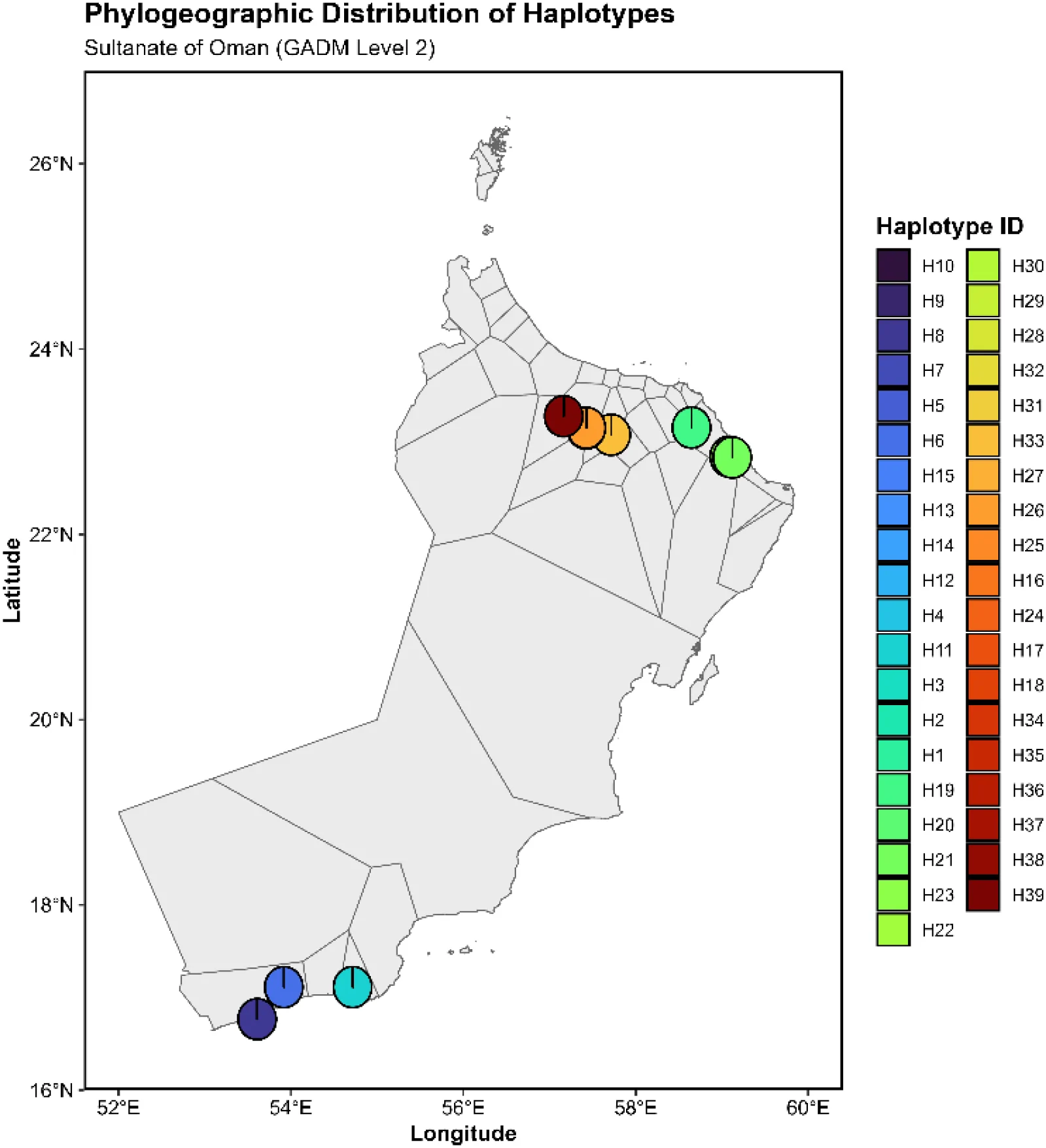
Phylogeographic structure of *Olea europaea* subsp. *cuspidata* in Oman. The figure shows the geographic distribution of cpDNA haplotypes based on GADM Level 2 administrative boundaries, with each pie chart representing a sampling locality and colors corresponding to specific haplotypes (H1–H39). The median-joining haplotype network illustrates the evolutionary relationships between these haplotypes, with circle sizes proportional to haplotype frequency and tick marks representing mutational steps.

Five chloroplast haplotypes were identified among the eight wild olive populations across Oman (**Figure 11**). Three distinct haplotypes were detected in the Dhofar mountain populations. Jabal Qara (DJQ) and Jabal Qamar (DJR) shared the same haplotype (Hap_1), while one accession from DJR represented a different haplotype (Hap_2), and all accessions from Jabal Samhan (DJS) formed a unique haplotype (Hap_3). Haplotype 4 (Hap_4) comprised five accessions from populations in the Eastern and Western Hajar Mountains, whereas one accession from Jabal Shams (WJS) formed a separate haplotype (Hap_5). The geographic locations of the cpDNA haplotypes and the median-joining network illustrating their relationships are presented in **Figure 12**.

## Discussion

This study provides a comprehensive assessment of the genetic diversity and population structure of wild olive (*Olea europaea* subsp. *cuspidata*) populations across Oman using single nucleotide polymorphism (SNP) markers generated from target capture sequencing with the Angiosperms353 probe set. SNPs are highly stable, abundant, and widely distributed throughout the genome, making them particularly powerful for detecting fine-scale genetic variation. Their use in this study offers valuable insights into the genetic makeup of the diversity within wild olive in Oman and provides a foundation for future conservation programs there. Such genomic information can facilitate the development and characterization of core collections based on adaptive genetic traits, which in turn will support the regeneration and sustainable management of *Olea* germplasm resources (Omire et al., 2022).

The results of the STRUCTURE, PCA, and DAPC analyses consistently identified two genetically distinct groups of wild olive samples across Oman. Accessions from the Dhofar Mountains in southern Oman formed Cluster I, whereas Cluster II comprised all accessions from the Eastern and Western Hajar Mountains. Evidence of admixture among individuals from the Eastern and Western Hajar populations indicates mixed ancestry and suggests some degree of genetic connectivity between these two regions. This pattern was also supported by the Neighbor-Net phylogenetic network, which showed overlapping relationships among accessions from both Hajar ranges. The close geographic proximity between the Eastern and Western Hajar Mountains (approximately 170 km apart) likely facilitates some level of gene flow (Besnard et al., 2007b). Olive pollen is known to disperse over long distances, even at regional scales of 200–300 km, as demonstrated by Fernández-Rodríguez et al (Fernández-Rodríguez and etal, 2014). in their study of airborne *Olea* pollen in the southwestern Iberian Peninsula. Although long-distance pollen dispersal has been documented in *Olea* species, such events are rare and unlikely to maintain effective contemporary gene flow between the Hajar and Dhofar populations.

In contrast, the wild olive populations in the Dhofar Mountains were genetically isolated from those in the Hajar Mountains (**Figures 4**-**12**). This strong differentiation can be attributed to the vast arid desert -part of the Rub’ al Khali (Empty Quarter)- that separates southern Oman from the northern mountain ranges which is a major biogeographical barrier. Beyond geographic distance alone, multiple environmental factors likely contribute to the observed north–south differentiation. The Rub’ al Khali desert not only acts as a physical barrier but also represents an extreme ecological gradient limiting both pollen and seed dispersal. In addition, differences in climatic conditions between the monsoon-influenced Dhofar region and the more arid northern mountains may further reinforce genetic divergence through local adaptation. Potential anthropogenic pressures, including land use changes and habitat fragmentation, may have also contributed to reducing connectivity among populations.

A similar pattern was reported by Besnard et al (Besnard et al., 2021). in North Africa, where the Saharan Desert limited gene flow between *O. europaea* subsp. *laperrinei* populations from Niger and Algeria, leading to significant genetic divergence. Comparable patterns of strong regional differentiation have also been documented in wild olive populations across East Africa and the Arabian Peninsula, suggesting that historical isolation and environmental heterogeneity have played a key role in shaping the evolutionary history of this subspecies (Kassa et al., 2019; Habib et al., 2021). Accordingly, within Oman, the wild olive populations of the Dhofar Mountains appear to be geographically and genetically isolated, with reduced gene flow both within and beyond this mountain range. This pattern is further supported by recent genomic evidence from Oman, which revealed clear genetic differentiation between wild and cultivated olive populations, highlighting strong genetic structuring in this region (Al-Yahyai et al., 2026). Such limited connectivity is consistent with previous studies showing reduced gene flow in fragmented and isolated populations compared to those that are spatially connected (Dzialuk et al., 2014; Serrote et al., 2022).

The deep genetic divergence observed at the regional level (PhiRT = 0.528) between the Hajar and Dhofar lineages suggests that the current population structure reflects historical habitat fragmentation rather than simple contemporary isolation by distance (IBD). While long-distance pollen dispersal has been documented in *Olea* species, such events are likely rare and insufficient to maintain effective gene flow across the vast Omani interior.

Palaeoenvironmental reconstructions for the Southern Arabian Peninsula (Preston et al., 2015) indicate a significant climatic shift toward aridity and increased regional dune emplacement between 5.9 and 5.3 ka cal. BP. This transition likely eliminated the lowland ‘corridors’ that once facilitated connectivity, effectively restricting *Olea europaea* subsp. *cuspidata* to high-altitude mountain refugia (Besnard et al., 2007b). Within these isolated ‘mountain islands,’ the cessation of migration allowed genetic drift to become the primary driver of population differentiation, shaping the distinct evolutionary lineages observed in northern and southern Oman today. These findings support a scenario in which historical connectivity was followed by fragmentation and long-term isolation, rather than ongoing gene flow between regions.

The results from PCA and DAPC analyses provided a slightly different pattern from the STRUCTURE analysis, revealing additional sub-structuring within the Omani wild olive populations. While STRUCTURE identified two main clusters corresponding to the Dhofar and Hajar mountain ranges, both PCA and DAPC suggested further genetic differentiation within the Hajar populations, indicating possible subgroups between the Eastern and Western Hajar ranges. Evidence of admixture was detected between individuals from these two regions, suggesting that genetic connectivity persists across the short distance separating them, consistent with patterns observed in wind-pollinated species (Buschbom et al., 2011; Chludil et al., 2025).

The fixation index (F_ST_) estimates the degree of genetic differentiation among populations. In this study, the overall F_ST_ value for wild olive populations within Oman was 0.3093, indicating moderate to high genetic differentiation among populations. According to Nassiry et al (Nassiry et al., 2009), F_ST_ values of 0-0.05 indicate low differentiation, 0.05-0.15 moderate, and values above 0.15 represent very high genetic differentiation. Consistent with these thresholds, differentiation was low within the Eastern and Western Hajar Mountains (F_ST_ = 0.00–0.05), but markedly higher between Dhofar and northern populations (F_ST_ = 0.38–0.43), confirming strong regional genetic structuring.

To clarify the relationship between differentiation metrics, it is important to distinguish between hierarchical levels of analysis. The global F_ST_ value (0.3093) is directly comparable to the AMOVA estimate at the population level (ΦST = 0.2814), and both indicate moderate to high genetic differentiation among populations. In contrast, the higher AMOVA value (ΦRT = 0.528) reflects deeper regional divergence between the northern Hajar and southern Dhofar lineages. Therefore, the apparent difference between F_ST_ and Φ values is expected and arises from their estimation at different hierarchical levels rather than inconsistency. Overall, both metrics consistently support strong population structuring driven by historical isolation.

Low F_ST_ values were observed among populations within the Eastern and Western Hajar Mountains, indicating limited genetic differentiation within each mountain range (**Figure 8**). In contrast, the Dhofar populations exhibited very high genetic differentiation (F_ST_ = 0.38–0.43) from both Hajar Mountain populations, confirming their genetic isolation. Isolation by distance (IBD) was detected across Oman; however, this pattern may reflect historical demographic processes rather than ongoing connectivity. This pattern aligns with previous studies demonstrating that geographic barriers can strongly structure genetic diversity in olive populations (Besnard et al., 2007a; Besnard et al., 2009; Levy et al., 2016; Grigoriadou Zormpa et al., 2025).

*Olea europaea* subsp. *cuspidata* is distributed across a large geographic range, from southern Africa to the Arabian Peninsula and extending to north-eastern Africa and China (Green, 2002). Population genetics theory generally predicts that species with a more localized geographic range tend to exhibit lower levels of genetic variation compared to widely distributed species (Cole, 2003; Su et al., 2025). However, numerous studies have reported that localized plant species can display similar or even higher levels of genetic variation than widespread species, indicating that additional factors may influence genetic diversity (Yoichi and Tomaru, 2014; Zhu et al., 2023). Based on the isolation-by-distance model, gene flow between distant populations of a widely distributed species is expected to be lower than between more closely situated populations of geographically limited species (Godt and Hamrick, 1998). In Oman, the average distance between populations in the Eastern and Western Hajar Mountains is 313 km, whereas Dhofar populations are separated by an average of 899 km. Correspondingly, genetic divergence is lower between the Eastern and Western Hajar populations (F_ST_= 0.00 to 0.05) and higher between the northern Hajar and Dhofar populations (F_ST_= 0.38 to 0.43). These results indicate that gene flow among populations in the northern mountains is greater than between Dhofar populations, reflecting the effect of geographic distance on genetic connectivity.

The observed genetic patterns in Omani wild olive populations are shaped by geographic isolation, habitat structure, and other ecological and demographic factors. The Dhofar populations are both geographically distant and isolated by extensive desert areas, which restrict pollen-mediated gene flow and seed dispersal, leading to high genetic differentiation (F_ST_= 0.38-0.43) and low connectivity with the northern Hajar populations. In contrast, the Eastern and Western Hajar Mountains are closer together and show evidence of admixture, consistent with greater gene flow and lower genetic differentiation (F_ST_= 0.00 to 0.05). Additionally, the heterogeneous topography and microclimatic variability within each mountain range may maintain localized genetic variation by creating sub-habitats that promote population persistence and adaptation. These results suggest that both geographic distance and habitat structure play central roles in shaping the population genetic structure of wild olives in Oman, highlighting the importance of considering these factors in conservation planning and germplasm management.

The high genetic differentiation revealed by DAPC, PCA, and STRUCTURE analyses indicates that wild olive populations in the Western and Eastern Hajar mountains are genetically close, whereas the Dhofar populations are highly isolated due to the vast desert (part of the Empty Quarter) separating them from the northern mountains. Negative F_IS_ values obtained for wild olive populations in the three mountain ranges (**Table 4**) are consistent with an outcrossing mating system, but are not necessarily indicative of restricted gene flow. Moderate to high genetic differentiation among populations (global F_ST_ = 0.3093) and low gene flow (Nm = 0.5584) suggest restricted connectivity among populations. This pattern is further supported by AMOVA results, which revealed that most genetic variation occurs within populations (71.86%), while a significant proportion is distributed among populations (28.14%; Φ_ST_= 0.2814, P = 0.001), consistent with the global F_ST_ estimate (0.3093). The mating system of *Olea europaea* subsp. *cuspidata* likely contributes to these patterns: it is a monoecious, diploid (2n = 46) species that outcrosses, is wind-pollinated, and its seeds are dispersed by small mammals and birds (Abiyu et al., 2015; Kassa et al., 2018). Clonal reproduction has not been observed in this subspecies, although related *O. europaea* subsp. *laperrinei* exhibits clonal growth (Baali-Cherif and Besnard, 2005).

The haplotype network based on plastid DNA SNPs identified five distinct wild olive haplotypes within Oman. The Eastern and Western Hajar mountains shared several haplotypes, reflecting gene flow and genetic connectivity between these northern populations, whereas the Dhofar populations exhibited unique haplotypes, highlighting their genetic isolation. Understanding the distribution of these haplotypes is essential for developing effective conservation strategies for wild olives in Oman. Knowledge of the genetic variation within and among populations can guide the establishment of core collections, inform habitat management, detect potential hybridization, and assess the adaptive potential of populations to environmental change (Hohenlohe et al., 2021).

From a conservation perspective, the strong genetic differentiation observed between northern and southern populations highlights the importance of region-specific management strategies. Dhofar populations should be considered a conservation priority due to their unique genetic lineages and geographic isolation. *In situ* conservation efforts should focus on the protection of natural habitats, particularly in the Dhofar Mountains, through habitat restoration and the establishment of protected areas. In addition, *ex situ* strategies, such as germplasm collection, seed banking, and breeding program development, are recommended to safeguard genetic resources and support future restoration efforts. These measures will help preserve the distinct evolutionary lineages of *O. europaea* subsp. *cuspidata* in Oman and maintain adaptive potential under environmental change.

## Conclusions

This detailed study provides essential insights into the genetic diversity and population structure of *Olea europaea* subsp. *cuspidata* across Oman. Wild olive populations exhibited relatively low genetic diversity within each mountain range but high genetic differentiation between the Dhofar mountains and both the Eastern and Western Hajar mountains (F_ST_ = 0.38–0.43; ΦST = 0.2814), indicating strong isolation of southern populations and potential risk of genetic erosion. STRUCTURE, PCA, and DAPC analyses consistently revealed two major genetic clusters: one comprising the Dhofar populations, which are genetically distinct, and the other including the Eastern and Western Hajar populations, which are more genetically connected. These findings highlight the uniqueness of the Dhofar populations and the high connectivity among northern populations, emphasizing the importance of region-specific conservation strategies. Based on our analyses, we recommend prioritizing germplasm collection from the genetically distinct Dhofar populations and implementing targeted protection measures for isolated Hajar populations. Such actions will help preserve the overall genetic variation of wild olive in Oman and provide practical guidance for establishing core collections, managing fragmented populations, and supporting conservation and breeding programs.

## Data Availability

The datasets presented in this study can be found in online repositories. The names of the repository/repositories and accession number(s) can be found below: https://www.ncbi.nlm.nih.gov/, SRR36345062–SRR36345109.
