# Correction: Genomic insights into population structure and conservation of wild olive (*Olea europaea* subsp. *cuspidata*) in Oman’s Dhofar and Hajar Mountains

**DOI:** 10.3389/fpls.2026.1963958

**Published:** 2026-08-17

**Authors:** Frontiers Production Office

**Affiliations:** Frontiers Media, SA, Lausanne, Switzerland

**Keywords:** genetic diversity, *Olea europaea* subsp. *cuspidata*, Oman, population structure, SNP markers, wild olive

The affiliation of Reviewer “Djamila Madani” has been corrected to “Mohamed Boudiaf University of M’Sila, Algeria”.

The original version of this article has been updated.

